# Humans expect generosity

**DOI:** 10.1038/srep42446

**Published:** 2017-02-14

**Authors:** Pablo Brañas-Garza, Ismael Rodríguez-Lara, Angel Sánchez

**Affiliations:** 1 Middlesex University London, Department of Economics, Business School, Hendon Campus, The Burroughs, London NW4 4BT, United Kingdom; 2 Grupo Interdisciplinar de Sistemas Complejos, Departamento de Matemáticas, Universidad Carlos III de Madrid, 28911 Leganés, Madrid, Spain; 3 Institute UC3M-BS of Financial Big Data, Universidad Carlos III de Madrid, 28903 Getafe, Spain; 4 Institute for Biocomputation and Physics of Complex Systems (BIFI), University of Zaragoza, 50018 Zaragoza, Spain

## Abstract

Mechanisms supporting human ultra-cooperativeness are very much subject to debate. One psychological feature likely to be relevant is the formation of expectations, particularly about receiving cooperative or generous behavior from others. Without such expectations, social life will be seriously impeded and, in turn, expectations leading to satisfactory interactions can become norms and institutionalize cooperation. In this paper, we assess people’s expectations of generosity in a series of controlled experiments using the dictator game. Despite differences in respective roles, involvement in the game, degree of social distance or variation of stakes, the results are conclusive: subjects seldom predict that dictators will behave selfishly (by choosing the Nash equilibrium action, namely giving nothing). The majority of subjects expect that dictators will choose the equal split. This implies that generous behavior is not only observed in the lab, but also expected by subjects. In addition, expectations are accurate, matching closely the donations observed and showing that as a society we have a good grasp of how we interact. Finally, correlation between expectations and actual behavior suggests that expectations can be an important ingredient of generous or cooperative behavior.

Humans are one of the four pinnacles of social evolution along with colonial invertebrates, social insects and nonhuman mammals^1^,^2^. Recent research points to psychological mechanisms, evolved to support our ultra-cooperative lifestyle, as the basis for human ultra-sociality^3^,^4^. Prominent among such mechanisms is that interaction with others sets up expectations. Expectations indeed grease the wheels of social integration. When facing others in a social context, we do not suppose that they will behave randomly, but rather we believe their actions will conform to our expectations for that context. In particular, expectations are deeply intertwined with cooperative and generous behavior: Thus, we expect dedication and care (beyond the pure delivery of services), for instance, when we visit the doctor or when we ask for advice in a shop. Crucially, this is also true of people whom we meet for the first time: without this sort of *wishful thinking*, we would probably not travel abroad, since there is always a risk of getting sick or needing help in different ways among strangers.

Besides affecting the emergence of social norms^5^,^6^ or the level of happiness^7^,^8^,^9^, expectations turn out to be crucial in many economic environments. They are associated with herding behavior^10^, decisions to trust in the investment game^11^, strategic thinking^12^,^13^,^14^, cooperation in social dilemmas^15^,^16^, ultimatum bargaining^17^ and many others. At the organizational level, employees’ expectations might affect their decisions on giving up their current job or accepting a particular offer, as expectations about peers’ performance influence their level of effort^18^. Expectations are indeed a well rooted concept in the setting of incomplete contracts, i.e., contracts that for several reasons fail to specify investment levels properly, or other contingencies. These type of relations can only work if the parties trust in the other’s performance^19^. Not surprinsingly, expectations have been taken as a reference point in many behavioral models^20^,^21^,^22^,^23^,^24^. However, little is known about people’s expectations of being treated generously and how such expectations relate to actual generous behavior.

In this paper we aim to answering the above questions by means of a comprehensive exploration of subjects’ expectations about generosity. An appropriate manner to study expectations in generosity is the dictator game (DG for short), which has provided a large body experimental evidence on altruistic behaviour in the lab during the last thirty years^25^,^26^. The DG is a simple one-shot game with two players: the first one (the dictator) is invited to divide a specified amount between himself and the second player (the recipient). The dictator may divide the pie in the manner he sees fit, while the recipient is not permitted to make any claim to the money. Theoretically, self-centered preferences predict that the dictator keeps all the pie and the recipient receives nothing; hence, any positive donation can be interpreted as proof of generosity. Contrary to the self-centered prediction, Engel’s meta-analysis^25^ shows that a huge number of individuals do offer nonzero, often sizeable portions of the pie to the recipient. On average, subjects donate between 20–30% of the total pie with a non-trivial fraction of subjects choosing an equal split. Interestingly, some authors argue that this is indeed a lower bound for generosity given the absence of social context within a lab experiment^26^,^27^,^28^,^29^,^30^,^31^.

Our specific goal here is to study if subjects expect this generous behavior in one-shot interactions, i.e., excluding any possible reciprocity effects^32^,^33^. Our study investigates also the accuracy of expectations; i.e., whether or not expectations are in line with the observed behavior. Previous research has focused on the relationship between the dictator’s expectations and his own behavior^34^,^35^,^36^ or the role of gender in expectations^37^. In order to provide a truly general insight on expectations of generosity, it is important to study as many relevant factors as possible. Towards this goal, we have designed and carried out a set of experiments in which subjects have to guess the donation that a dictator has already given in a DG. We cover a wide range of conditions by varying the degree of involvement, the social distance, the role of the guesser, the possibility of hedging, the size of the stake or the location of the experiment. Although these elements have been found to affect donations in a DG^25^,^26^,^27^,^28^,^29^,^30^,^31^,^38^, there is yet no systematic investigation of how they could possibly influence expectations about generosity.

Our research questions and their corresponding experimental conditions are summarized in Table 1 (see the Methods for a full description of the corresponding experimental setups). Our elicitation covers subjects’ expectations about the donation they expect to receive (with the usual or higher stakes), the donations others are going to receive (lack of involvement in the outcome), and the donations from absent dictators or from dictators from a previous experiment (thus probing the effects of social distance between subjects). All choices are incentivized (subjects receive monetary payments according to the accuracy of their predictions). To avoid hedging, we consider a condition in which external observers do not receive the dictator’s donation, but are paid the show-up fee plus an additional amount for their correct guesses^39^. Finally, we also asked dictators to guess the donations of other dictators, and hence there is possible influence of one’s own choice in the answer.

## Results

**Figure 1:**
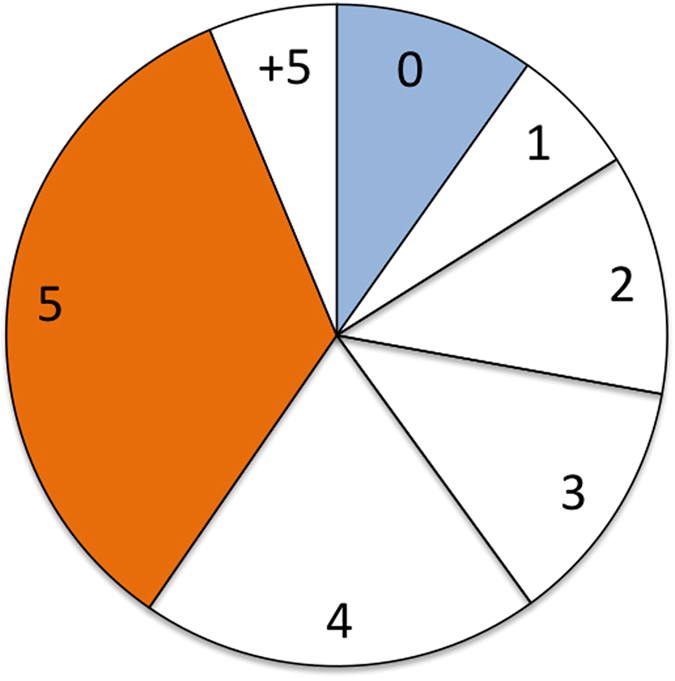
Distribution of guesses aggregated over all experimental conditions (255 observations). Subjects seldom predicts selfishness (in blue). The modal expectation (in orange) is hyper-fair behavior, i.e., an equal split of the pot. A total of 25 guesses (10%) correspond to selfish behavior while 87 guesses (34%) correspond to the equal split.

**Figure 2:**
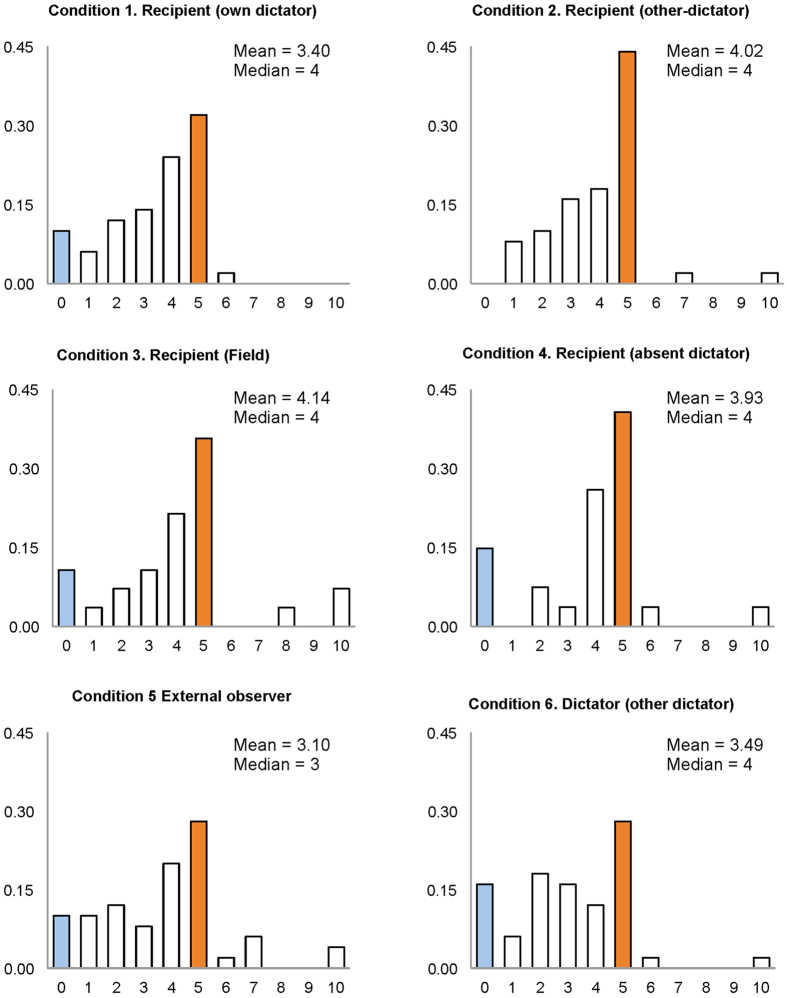
Distribution of guesses across conditions. Hyper-fair behavior (50–50) is the modal expectation (in orange) across conditions; pure selfish behavior (in blue) is barely predicted. There are no significant differences across conditions.

The main result of our study is that the majority of people expect generous behavior with the modal prediction being the hyper-fair outcome; i.e., the equal split. Figure 1, aggregates results for all six conditions studied, and Fig. 2 shows the distribution of guesses for each condition along with the mean and median expectation in each condition. It is very clear from the plots that, both in the aggregate and across conditions, subjects expect not only generosity (meaning positive donations), but large positive donations close to hyper-fair behavior from dictators, and that the distribution of guesses is roughly the same in all cases. It is remarkable that the largest fraction of subjects expect the equal split. Interestingly, a significant fraction of subjects expect a donation of 4, which is the median in all the conditions except Condition 5 (observer guessing a previous donation). Overall, 60% expect a donation of 4 or more, which is a large majority. As regards strictly selfish behavior, we observe that it is predicted by roughly 10–15% of subjects, with the exception of condition 2, where the recipient has to make a prediction about another dictator: in this condition, none of our subjects predicted 0. On the other hand, subjects seldom predict donations above the equal split. However, in every condition -except condition 1, guessing what one is going to receive— there is at least one subject who predicts full donation.

When we look at the factors that may affect expectations, the Kruskal-Wallis test cannot reject the null hypothesis that all guesses come from the same distribution at any common significance level, under different assumptions (*p* > 0.173). Pairwise comparisons confirm that there are not significant differences between the underlying distributions of any two conditions (*p* > 0.305) (see Table S1 and the discussion in the Supplementary Information).

An econometric analysis confirms that generous behavior is expected regardless of the location, the degree of involvement in the outcome, the social distance or the size of the stakes. Table 2 reports the estimates of four different specifications that attempt to predict what subjects expect that dictators will donate. These specifications are frequently used to model the dictator’s behavior^25^. We first considered an OLS regression, but because donations cannot be smaller than 0 or larger than 10 (cf. Figs 1 and 2), one may argue that the data are censored. In that case, we included a Tobit model as it may be more appropriate. Subsequently, we studied a hurdle model, that also accounts for the “spike” in the zero donation, but assumes that the forces affecting the willingness to guess a positive donation may differ from the ones that determine what subjects expect dictators to donate. Such a hurdle specification therefore assumes that subjects have to decide whether to guess any donation at all with a logit model (Hurdle0), and only then the process determining the positive guessing applies (Hurdle+). In line with our discussion so far, guesses are found to be consistent across conditions, as none of the dummy variables are significantly different from zero. As can be seen from the Table 2, for OLS and Tobit models, the value of the constant is significantly different from zero, which indicates that subjects expect a positive donation from the dictator. The negative (and significant) value of the constant in Hurdle0 can be interpreted as subjects not being likely to predict the zero donation.

**Figure 3:**
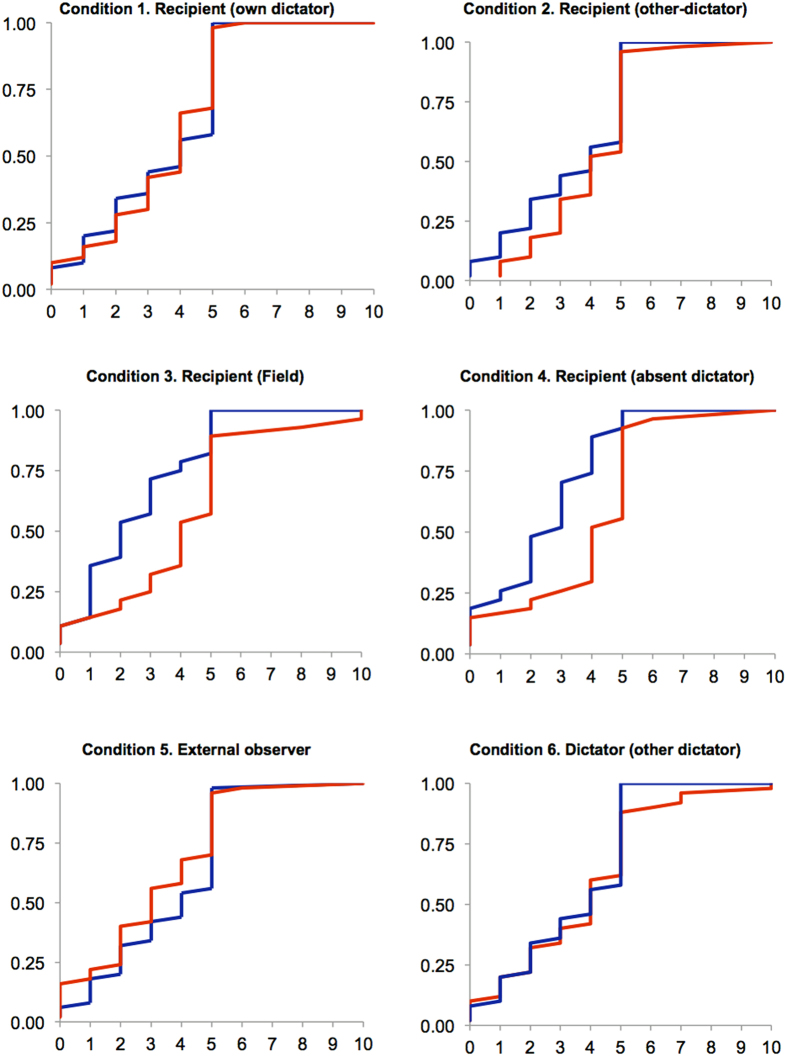
Expectations and observed behavior across conditions. Cumulative distribution of guesses (red lines) and dictators’ donations (blue lines) in each condition. Expectations are very accurate, in particular in conditions 1, 2, 5 and 6.

Next, we analyze the accuracy of expectations by comparing the elicited beliefs with the actual donation of dictators. Figure 3 presents our data using the cumulative distribution of guesses and donations in each condition. Subjects turn out to be quite accurate in their predictions in Conditions 1, 2, 5 and 6, where we find no significant difference between the expected behavior and actual donations (*p* > 0.130). In the presence of high incentives (condition 3) or when dictators are absent (condition 4), recipients tend to overestimate the amount they are going to receive from dictators (*p* < 0.01). As we have discussed above, expectations are the same in all conditions. Hence these disagreements arise from the fact that dictators are more selfish in conditions 3 and 4 (see Fig. S1 in the Supplementary Information). This is in line with previous evidence suggesting that dictators donate less in the presence of high stakes^25^,^26^,^38^ or when there is no direct contact between dictators and recipients^27^,^28^,^29^.

**Figure 4:**
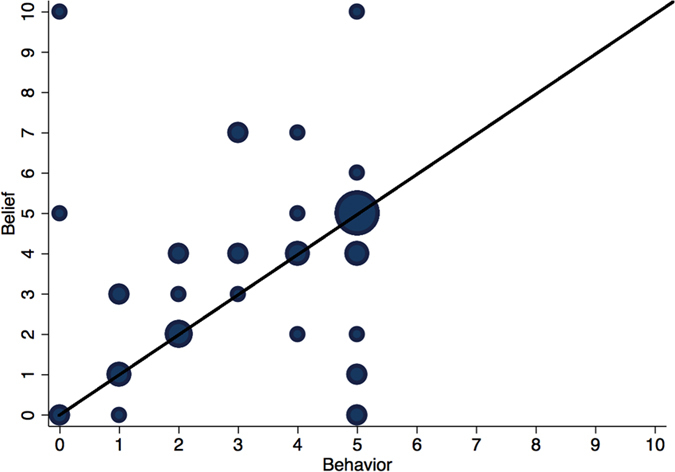
Within-subjects analysis of expections and own behavior (Condition 6). Experimental subjects’ behavior is correlated with their expectations about others’ donations. The size of the circles is proportional to the number of subjects with a given belief and behavior.

Finally, we look into the relationship between a subject’s behavior and her own expectation. Our data from condition 6 (where dictators’ expectations about others’ donations were elicited) provide us with the results depicted in Fig. 4. We observe a clear correlation between the dictators’ donations and their beliefs about how other dictators would behave (*r*^2^ = 0.28, *p* = 0.046; when restricted to positive donations only, *r*^2^ = 0.40, *p* = 0.005). The fact that half of dictators donated an amount equal to their belief highlights the deep connection between expectations and behavior^34^,^35^,^36^. On the other hand, 20% (30%) of subjects expected less (more) than their own donation, as can be observed from the circles above (below) the diagonal in Fig. 4.

## Discussion

In summary, our series of experiments strongly supports the conclusion that subjects expect generous behavior in situations, such as those modeled by the DG, where self-interest should be the rule. Our findings are derived in one-shot games, i.e., in the absence of any expectations of reciprocity. This is a clear indication that humans expect other humans to behave socially. Importantly, expectations are well connected to the degree of generosity and are not affected by the degree of involvement, the social distance, the possibility of hedging, the size of the stake or the location of the experiment.

We believe that our results are related to the experimental evidence showing that cooperation might be the default option for a large fraction of the population^40^,^41^,^42^. Indeed, in one-shot or in the first round of iterated Prisoner’s Dilemma or Public Good games approximately half of the subjects cooperate^43^. Remarkably, the fraction of people is very similar to the fraction of subjects expecting hyper-fair offers in our experiments. Current evidence suggests that cooperative choices are correlated with generosity^44^,^45^. We have seen that expectations about generosity are also correlated with generous behaviour, what might indicate a common prosocial motivation towards cooperation.

The findings we have reported suggest an important direction for future work, namely whether expectations in one game (or strategic situation, generally speaking) carry over to a different one. Recent experiments by Peysakhovich *et al*.^46^ suggest a sizable fraction of the population may exhibit a ‘cooperative phenotype’, leading them to make prosocial decisions across games. Studying the relationship between expectations and these phenotypes is likely to lead to a breakthrough in the understanding of cooperation and, above all, in providing solid indications as to how to promote prosocial behavior.

From a broader perspective, the so-called Neo-Darwinian theory^47^ suggests that altruism may be detrimental as it reduces the one’s fitness while enhancing the fitness of others. Arguably, altruism may have positive effects from an evolutionary viewpoint, as human beings are characterized by bounded rationality and may learn from other individuals what is good for them^48^,^49^. While there might be different mechanisms to sustain altruism and cooperative behaviour (e.g., punishment^15^,^50^,^51^), we argue that expectations might be another important factor driving altruism and social norms. Fair behavior might be well-internalized and thus becomes the de facto rule, which is then reflected in subject expectations and leads to generous behavior. Key for this mechanism to work is the accuracy of the beliefs held, as we have seen we are able as a society to have a clear idea of what to expect from others. Recent findings highlight that subjects keep believing in prosocial behaviour in repeated contexts, even when cooperation effectively decreases^52^. Further research on the connection between expectations and own behavior (including the possibility of a casual relationship between the two), on the existence and characteristic of cooperative phenotypes, and on the accuracy of expectations is needed to shed light on these issues.

## Methods

This section explains the different conditions used along this research, the research questions and the experimental procedures followed in each stage. An English translation of the instructions used in the experiments are included in the Supplementary Information. The data comes from 205 subjects who made a total of 255 (incentivized) guesses about the dictator’s donation (note that 50 subjects made two guesses as they participated in conditions 1 and 2).

An informed consent form was signed by all subjects taking part in the experiment. Anonymity was always preserved (in agreement with Spanish Law 15/1999 on Personal Data Protection) by randomly assigning a numerical code to identify the participants in the system. No association was ever made between their real names/addresses and the results. As is standard in socio-economic experiments, no ethic concerns are involved other than preserving the anonymity of participants. This procedure was checked and approved by the Vice-dean of Research of the School of Economics of the University of Granada; the institution hosting the experiments. All methods were performed in accordance with the relevant guidelines and regulations.

### Conditions 1–2: Recipients in the lab guessing own and others’ donations

A total of 100 subjects, all of them undergraduate students from fields other than Economics and Business, reporting no previous experience in experiments, participated in an experiment at the Laboratory for Research in Experimental Economics (LINEEX), University of Valencia, in February 2013. The experiment was conducted using the z-Tree software^53^. Subjects were randomly assigned to the role of dictator or recipient. Following standard instructions, dictators were asked to make a division of the pie (10 Euros) in integer numbers. The instructions (read aloud by the instructor) made subjects aware that keeping the whole pie was acceptable. Once the dictators had reached their decision, the recipients (*n*_1_ = 50) were privately asked to guess the donation they were going to receive. A scoring rule with monetary incentives motivated recipients to make accurate guesses: Subjects were paid 5 Euros for correct answers, 1 Euro if they failed by just one unit, and 0 otherwise. While the dictators made their decisions, the recipients (*n*_1_ = 50) were privately asked to guess the donation they were going to receive (Condition 1) as well as the donation made by *another* randomly selected dictator in the room (*n*_2_ = 50, Condition 2). Order effects were controlled for (i.e., half of them first made the guesses for their own dictators). No order effect was found; the distribution of guesses of those recipients who estimated the donation of their dictator first is not different from those who estimated other dictators first (Mann-Whitney U or the t-test, p-values > 0.183). At the end one of the beliefs (Condition 1 or 2) randomly selected was paid out. Recipients received this amount in addition to the donation of their matched dictator (see final remarks). Subjects earned on average 8 Euros for the 30 minute session, including the show-up fee of 2 Euros.

### Condition 3: Recipients in the field guessing own donations

This experiment was run at the Universidad Autonoma de Baja California Sur (UABCS) at La Paz (Mexico) in 2006. This location was chosen for two main reasons. First, to the best of our knowledge, no one had ever run any experiments at that location; therefore the whole population was completely inexperienced. Second, there was an interest in exploring the effect of “high stakes” on expected generosity. Thus, the size of the surplus to be divided (200 pesos ≈15 US$, ≈14 Euros in 2006) was enough to buy 25 beers at any canteen there at La Paz. This would have cost more than $50 in the US in 2006 (this amount more than triples the standard pie of $10 in the DG). A total of 56 students were recruited the week prior to the experiment. On the day of the experiment, subjects waited in the central plaza of the school near the auditorium. Twenty-eight subjects were randomly selected as dictators (*n*_3_ = 28), while the remaining subjects were asked to wait for 15 minutes. Dictators received a package comprising a large brown envelope with another smaller white envelope inside, containing ten 20-mexican peso bills (200 pesos) and experimental instructions. Instructions stated that the money they wished to keep should be placed within the small white envelope and then in their pockets. The money they wished to donate to the recipients waiting outside had to remain in the big envelope. When recipients were asked to come in, dictators left by the back door, making communication among them impossible. Each recipient was seated 2 meters away from the place where their particular dictator had been seated and left the big envelope. Recipients received the instructions that their corresponding dictators had left. It was explained that these instructions belonged to the previous participants and then read them aloud. Recipients were informed that they would definitely receive the money in the envelope. They could earn 80 additional pesos if they guessed correctly the number of bills in the envelope, 20 pesos if they failed by just one unit, and 0 additional pesos otherwise. Average earnings were 150 pesos (≈12 US$, ≈10 Euros) in this condition.

### Condition 4: Recipients in the lab paired with absent dictators

A total of 27 students at the University of Granada were recruited by standard procedures in May 2008. When subjects arrived at the lab they found the experimental instructions and envelopes containing the donations of dictators of a previous experiment^54^. Again, subjects were asked to guess the donation contained in the envelope using the same scoring rule as in Conditions 1 and 2. Recipients received this amount in addition to the dictator’s donation in the envelope. Data from this condition differs from previously collected data in that dictators were absent when recipients made their prediction (i.e., recipients did not see any dictator in the room, nor did they receive any information about them).

### Condition 5: External observers guessing dictators’ donations

One week after the experimental sessions ran in the LINEEX (see Conditions 1 and 2) 50 new subjects were recruited. They received the instructions of the game (read aloud) in Condition 1 and were asked to predict dictators’ behavior, that is, donations to recipients in the experiment one week before. Participants were asked to guess the amount donated by a randomly selected dictator. They were informed that they would not receive the dictator’s donation. In line with all previous conditions, subjects were given incentives to make accurate guesses. The same scoring rule was used as before (5 Euros for a correct guess, 1 Euros if they failed by just 1 unit and 0 otherwise). The observations for this condition correspond to external observers. As in the case of Condition 2, this should allow us to explore the role played by involvement in the outcome.

### Condition 6: Dictators guessing the donation of other dictators

Dictators in Condition 1 (*n*_6_ = 50) were invited to make a second decision after dividing the pie. They had to predict what another dictator in the same area had donated to his or her corresponding recipient. Again, we use the same scoring rule with monetary incentives (5 Euros if they are perfectly accurate, 1 Euro if they fail by one and zero otherwise) to motivate dictators to make accurate guesses. Dictators received this amount in addition to that which they decided to keep in the DG.

### General comments for all conditions

Recipients in Conditions 1, 2, 3 and 4 were rewarded for their guesses, and received this amount in addition to the donation of their matched dictator. Although there is not much evidence for hedging strategies^39^, recipients may have incentives to hedge in these conditions. Clearly, hedging is not possible in Conditions 5 or 6, where dictators’ donations are not being received by guessers. It was decided not to use a payment scheme to avoid hedging in conditions 1 to 4 (e.g., paying recipients only once -i.e., either the dictator’s donation or one of their guesses) because it would imply deception against the dictator (who made a donation thinking that a recipient would receive the money).

Although we incentive beliefs in a quite standard manner, we acknowledge that our incentive scheme does not allow for eliciting the whole distribution of beliefs. Instead, we may be eliciting something closer to the modal expectation of subjects. In this regard, we could have used other methods for eliciting beliefs, which also come at the cost of making some assumptions; e.g., about risk preferences (for different methods and problems to elicit beliefs see refs 55, 56, 57).

Finally, it was important that dictators make their decision about donations without knowing that recipients in the experiment would make guesses about donations, thus avoiding any strategic giving. Along these lines, we deliberately decided to elicit dictators’ beliefs after they made their donation to eliminate any *focusing* influence as asking subjects about others’ behavior before playing the DG might trigger pro-social behavior^35^.

### Behavior across conditions

The critical difference between Conditions 1 and 2 is that the recipient should feel less involved in the latter. Since they are not guessing the money they are going to receive but the donation to a third person, less wishful thinking is expected. Using Conditions 1 and 2, we can therefore see if recipients overestimate (or underestimate) the amount of money they are going to receive compared with what they believe other recipients will get. We can see if the fact of being involved in the outcome has some effect on expected generosity, as it is the case when dictators make donations for themselves or for others^58^,^59^. The intention of Condition 3 is to assess the importance of the *lab* effect on expected generosity. Another interesting feature of this condition, apart from introducing high stakes, is that recipients received the instructions once dictators left the room. This is not the case with previous conditions, under which instructions are read aloud in front of dictators and recipients (i.e., in Conditions 1 and 2 some credibility issues are minimized). This issue is further explored under Condition 4, where recipients guess the donation of an absent dictator. It is important to emphasize that while wishful thinking remains intact in Condition 4 - since the subjects are recipients of the money- the social distance is maximized^60^ since the dictators who did the job were absent when recipients made their guesses. Interestingly, Condition 5 can be interpreted as an extreme variation of Condition 4. In both cases, the dictator is absent but, on top of that, subjects who make their guesses are not going to receive the dictator’s donation in Condition 5. Any wishful thinking is therefore eliminated. Note that hedging is not possible in this condition. Finally, Condition 6 provides us with new evidence: since these participants were dictators themselves and had already divided the pie, they may have felt that they had some *property rights* (i.e., “owing” “the game”) and therefore might be more likely to predict selfish behavior. Because they were not receiving any donation, apart from what they decided to keep, dictators should not have suffered any wishful thinking either.

## Additional Information

**How to cite this article**: Brañas-Garza, P. *et al*. Humans expect generosity. *Sci. Rep.* **7**, 42446; doi: 10.1038/srep42446 (2017).

**Publisher's note:** Springer Nature remains neutral with regard to jurisdictional claims in published maps and institutional affiliations.

## Supplementary Material

Supplementary Information

## Figures and Tables

**Table 1:** Summary of questions addressed in this study and the corresponding experimental conditions.

	Question	Condition
1	Do experimental subjects in the lab expect selfish behavior?	Recipient guessing the *donation she is going to receive* in a lab experiment
2	Do experimental subjects in the lab expect selfish behavior when they are not involved in the outcome?	Recipient guessing the donation *other recipient* is going to receive in a lab experiment
3	Do experimental subjects (in the field) expect selfish behavior in the presence of high stakes?	Recipient guessing the donation she is going to receive in a field experiment with *high incentives*
4	Do experimental subjects in the lab expect selfish behavior when dictators are absent?	Recipient guessing the donation she is going to receive from an *absent dictator*
5	Do experimental subjects in the lab expect selfish behavior when they are just observers?	A third party (observer) guessing the donation that a recipient has received in a *previous experiment*
6	Do experimental subjects in the lab expect selfish behavior after they divided the pie?	A *dictator guessing* the donation of other dictator

Note: Subjects have to guess the dictator’s donation in the DG. Across conditions, we vary the degree of involvement, the social distance, the role of the guesser, the possibility of hedging, the size of the stake or the location of the experiment. We can therefore assess how these features affect expectations about generosity in one-shot interaction with strangers.

**Table 2:** Econometric results for guesses about the dictator’s donation.

	OLS (1)	Tobit (2)	Hurdle0 (3)	Hurdle+ (4)
C2 (Other dictators)	0.620 (0.41)	0.717 (0.44)		0.102 (0.42)
C3 (Field)	0.743 (0.48)	0.747 (0.53)	0.077 (0.77)	0.355 (0.50)
C4 (Absent dictator)	0.526 (0.49)	0.489 (0.53)	0.448 (0.72)	0.342 (0.52)
C5 (Observer)	−0.300 (0.41)	−0.368 (0.45)	0.539 (0.61)	−0.037 (0.44)
C6 (Dictator)	0.320 (0.29)	0.325 (0.31)	0.000 (0.47)	0.149 (0.31)
Constant	3.400** (0.29)	3.303** (0.32)	−2.197** (0.47)	−0.499 (0.31)
*n*	255	255	205	230

Note: Robust standard errors in parentheses. The hurdle model considers 205 observations because recipients never predict that other dictators will donate zero; i.e., Condition 2 is not taken into account in the analysis. Hurdle+ relies on the 230 observations that correspond to positive guesses. Significance at the *5%, **1% level. We observe that subjects expect for dictators to donate a positive amount. There are no differences across conditions therefore the degree of involvement, the social distance, the role of the stakes do not influence the degree of expected generosity.
