# Supplementary material for: Humans expect generosity
Source: Sci Rep. 2017 Feb 14;7:42446. doi: 10.1038/srep42446 (PMC5307963; doi:10.1038/srep42446)
Supplement: Supplementary Information [file srep42446-s1.pdf]

Supplementary Information for

Humans expect generosity

Pablo Brañas-Garza,[1,*] Ismael Rodríguez-Lara, [1], Angel Sánchez [2,3,4]

[1]Middlesex University London, Department of Economics,
Business School, Hendon Campus, The Burroughs,
London NW4 4BT, United Kingdom
[2]Grupo Interdisciplinar de Sistemas Complejos, Departamento de Matemáticas,
Universidad Carlos III de Madrid, 28911 Leganés, Madrid, Spain
[3]Institute UC3M-BS of Financial Big Data, Universidad Carlos III de Madrid,
28903 Getafe, Spain
[3]Institute for Biocomputation and Physics of Complex Systems (BIFI), University of Zaragoza,
50018 Zaragoza, Spain

[*]To whom correspondence should be addressed; E-mail: branasgarza@gmail.com

Table 1 in the main text displays the distribution of expected behavior across conditions. Table S1 below presents an overview of the main statistics in each condition. We can test whether all guesses come from the same distribution using a Kruskal-Wallis test. This cannot reject the null hypothesis of equality of distributions at any common significance level, both when we assume that guesses from C1 and C2 are unpaired (p-value = 0.199) and when we exclude any of the two conditions from the analysis (p-values = 0.173 and 0.287, when we exclude C1 and C2 respectively).

We have performed pairwise comparisons using a Mann-Whitney test (a Wilcoxon signed-rank test for paired samples is used to compare Condition 1 and 2). Because multiple tests were performed, the p-values (reported in Table S2) are corrected to control the familywise

error rate using the Holm-Bonferroni method (*1, 2*). The results suggest that there are not statistically significant differences between the underlying distributions of any two conditions at any common significance level (p-values > 0.305).

In order to assess whether or not expectations about generosity are accurate, we need to compare them with the actual behavior of dictators. Fig. S1 displays the distribution of donations in all the conditions. While we observe that dictators are generous, the Kruskal-Wallis test suggests that at least one sample stochastically dominates one other sample ($\chi^2_2 = 7.11$, p= 0.028). Pairwise comparisons across conditions confirm that donations are smaller in Conditions 3 and 4 ($p < 0.021$) indicating that dictators are more selfish when stakes are high or there is social distance (*3–5*). These findings, in turn, imply that subjects overestimate the generosity of dictators in these conditions, while they are quite accurate in the rest of conditions. The p-values when comparing the distribution of guesses and donations using the Mann-Whitney test are as follows. Condition 1: $p = 0.844$; Condition 2: $p = 0.182$; Condition 3: $p = 0.0090$; Condition 4: $p = 0.003$; Condition 5: $p = 0.322$; Condition 6: $p = 0.130$.

Table S1: Summary of the data

Condition		n	Mean	Median	Min.	Max.	Observed frequency Guess = 0	Guess = 5
C1. Recipient	(own-dictator)	50	3.40	4	0	6	10%	32%
C2. Recipient	(other dictator)	50	4.02	4	1	10	0%	44%
C3. Recipient	(field)	28	4.14	4	0	10	11%	36%
C4. Recipient	(absent dictator)	27	3.93	4	0	10	15%	41%
C5. External	(observer)	50	3.10	3	0	10	16%	28%
C6. Dictator	(other dictator)	50	3.40	4	0	10	10%	32%

Note: The modal value was 5 in every single condition.

Table S2: Pairwise comparison of distributions across conditions.

	C2 (other dictator)	C3 (field)	C4 (absent dictator)	C5 (observer)	C6 (dictator)
C1 (own-dictator)	1.728	1.146	1.212	0.934	0.510
C2 (other dictator)		0.005	0.059	2.320	0.848
C3 (field)			0.017	1.750	0.630
C4 (absent dictator)				1.724	0.576
C5 (observer)					1.272

Notes: The comparisson between C1 and C2 is a within-subject (i.e., we consider the Wilcoxon signed-rank test). Because we perform a total of 15 pairwise comparissons, we correct the p-values using the Holm-Bonferroni method. The smallest of the unadjasted p-values is 0.020 (when comparing C2 and C5). This is not smaller than $\alpha/15$ so we fail to reject the null hypothesis that guesses in C2 and C5 come from the same distribution at any common significance level α =0.10,0.05,0.01.The rest of the null hypotheses are therefore rejected.

References

1. Bonferroni, C.E.. Il calcolo delle assicurazioni su gruppi di teste. Tipografia del Senato (1935).

2. Holm, S., 1979. A simple sequentially rejective multiple test procedure. *Scandinavian Journal of Statistics* **6**, 65-70 (1979).

3. Engel, C., 2011. Dictator games: A meta study. *Experimental Economics* **14**, 583-610 (2011).

4. Cooper, D.J., & Kagel, J.H., 2012. Other-regarding preferences: A selective survey of experimental results in *Handbook of experimental economics II* (eds. Kagel, J.H., & Roth, A.E.) (Princeton University Press, Princeton, NJ, 2012).

5. Novakova J, & Flegr J. How much is our fairness worth? The effect of raising stakes on offers by proposers and minimum acceptable offers in dictator and ultimatum games. PloS ONE **8**, e60966 (2013).

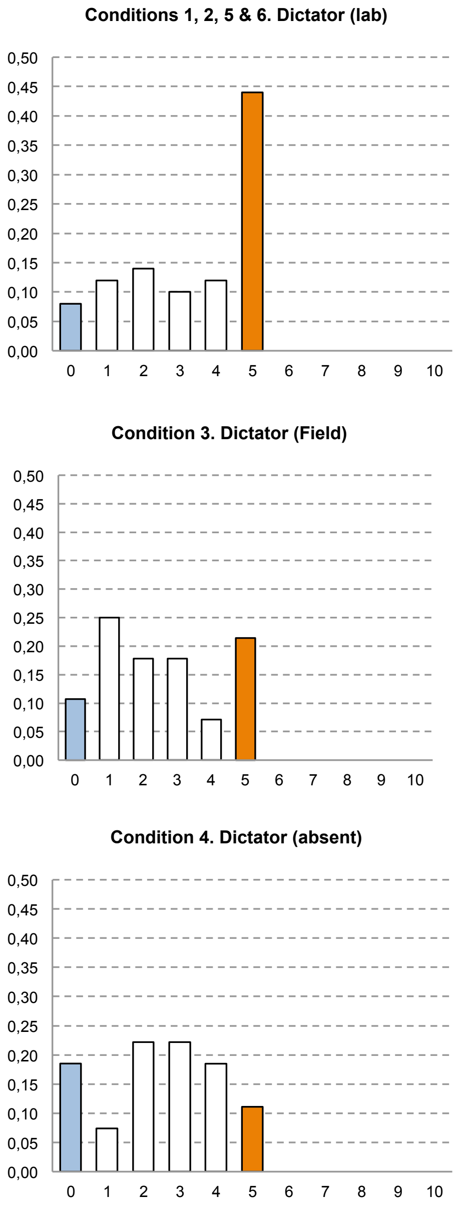

Figure 1: **Experimental subjects are generous and donate positive amounts but we observe an effect of high-stakes and social distance.** Histograms of the donations by dictators in the different conditions.
